# The Role of Long Noncoding RNAs in Human Papillomavirus-associated Pathogenesis

**DOI:** 10.3390/pathogens9040289

**Published:** 2020-04-15

**Authors:** Surendra Sharma, Karl Munger

**Affiliations:** Biochemistry Program, Graduate School of Biomedical Sciences; Department of Developmental, Molecular and Chemical Biology, Tufts University School of Medicine, Boston, MA 02111, USA; Surendra.Sharma@tufts.edu

**Keywords:** human papillomavirus, viral oncogenesis, cervical carcinoma, lncRNA, E6, E7

## Abstract

Infections with high-risk human papillomaviruses cause ~5% of all human cancers. E6 and E7 are the only viral genes that are consistently expressed in cancers, and they are necessary for tumor initiation, progression, and maintenance. E6 and E7 encode small proteins that lack intrinsic enzymatic activities and they function by binding to cellular regulatory molecules, thereby subverting normal cellular homeostasis. Much effort has focused on identifying protein targets of the E6 and E7 proteins, but it has been estimated that ~98% of the human transcriptome does not encode proteins. There is a growing interest in studying noncoding RNAs as biochemical targets and biological mediators of human papillomavirus (HPV) E6/E7 oncogenic activities. This review focuses on HPV E6/E7 targeting cellular long noncoding RNAs, a class of biologically versatile molecules that regulate almost every known biological process and how this may contribute to viral oncogenesis.

## 1. Human Papillomaviruses as Oncogenic Drivers

Papillomaviruses are a large family of non-enveloped viruses with ~8000 base pair, circular, double stranded DNA genomes. They have been detected in almost all vertebrates, are highly host-specific and preferentially infect squamous epithelial tissues. More than 440 human papillomaviruses (HPVs) have been molecularly characterized as of 03/2020, and they are organized into five phylogenetic genera: alpha, beta, gamma, mu and nu [1]. HPVs exhibit a marked preference for infecting specific squamous epithelial tissue types; most alpha HPVs infect mucosal epithelia, whereas beta, gamma, mu and nu HPVs preferentially infect cutaneous epithelia. HPV infections are either asymptomatic or cause formation of generally benign hyperplastic lesions, or warts. Some cutaneous HPV infections contribute to initiation of cutaneous squamous cell carcinomas, particularly in long-term immunosuppressed organ transplant patients, and in individuals with a rare hereditary skin disease, epidermodysplasia verruciformis [2,3]. The mucosal alpha HPVs can be clinically classified into low and high-risk types. Low-risk HPVs cause benign genital warts, whereas high-risk HPVs cause premalignant lesions that can progress to carcinomas. Approximately 5% of all human cancers are caused by high-risk HPV infections. These include almost all cervical carcinomas, a large fraction of other anogenital tract carcinomas and a growing percentage of oral cancers, particularly oropharyngeal carcinomas [4]. 

High-risk HPV-associated cancers are generally non-productive infections and only two viral genes, E6 and E7, are consistently expressed. HPV E6 and E7 encode low molecular weight, cysteine-rich, zinc-binding proteins of ~150 and ~100 amino acids, respectively. Despite their diminutive size, they are potent oncogenic drivers and are necessary for tumor initiation, progression and maintenance. They lack intrinsic enzymatic activities and do not directly bind to specific DNA sequences. Hence, they function by binding to host cellular regulatory molecules, thereby subverting their normal physiological activities [5,6]. As a consequence, HPV E6 and E7 target almost all cellular processes that have been designated “hallmarks of cancer” [7,8]. A large number of cellular protein interaction targets for E6 and E7 have been identified, most prominently the TP53 and retinoblastoma (RB1) tumor suppressor proteins, respectively [9,10]. Similarly, dysregulation of the cellular transcriptome by E6 and E7 has been amply documented but the majority of studies have focused on enumeration of the expression profiles of protein-encoding mRNAs. Given, however, that ~98% of the cellular transcriptome does not encode proteins, a significant amount of information has remained untapped.

The majority of studies on the contributions of noncoding RNAs to HPV carcinogenesis has focused on one class, the microRNAs (miRNAs) [11]. However, more recently there has been an emerging interest in determining the mechanistic contributions of another, large class of noncoding RNAs, the long noncoding RNAs (lncRNAs), in the context of HPV-associated carcinogenesis.

## 2. Long Noncoding RNAs

Long noncoding RNAs (lncRNAs) are defined as transcripts of >200 nucleotides with no or limited coding potential of <100 amino acids. Large intergenic noncoding RNAs (lincRNAs) are a subset of lncRNAs that do not overlap with protein coding genes, whereas other lncRNAs share some overlap, either on the sense or antisense strand, with coding genes [12]. The first cellular lncRNAs, H19 and X-Inactive Specific Transcript (XIST), were discovered in the early 1990s [13,14]. With the development of high-throughput sequencing techniques in the late 2000s, there was substantial increase in identified lncRNAs. Compared to the ~21,000 protein coding genes, the number of lncRNA genes has been estimated to be in the range of ~15,000 to ~58,000 [15,16]. As sequencing depth increases, it is expected that additional lncRNAs will be identified. The majority of lncRNAs are transcribed by RNA Polymerase II, have 5′ cap structures and are 3′ polyadenylated, rendering them biochemically indistinguishable from mRNAs. LncRNAs can localize to nuclear as well as cytoplasmic compartments. 

Only ~20% of lncRNA nucleic acid sequences are significantly conserved between humans and mice, whereas the remaining lncRNAs only share small areas of microhomology [17]. The fact that such microhomologies are significant has been impressively demonstrated by complementation experiments. For example, despite limited sequence similarity of the linc-birc6 (megamind) and linc-oip5 (cyrano) lncRNAs across species, the phenotype of megamind and cyrano depletion in zebrafish was rescued by expression of murine or human transgenes [18].

**Figure 1:**
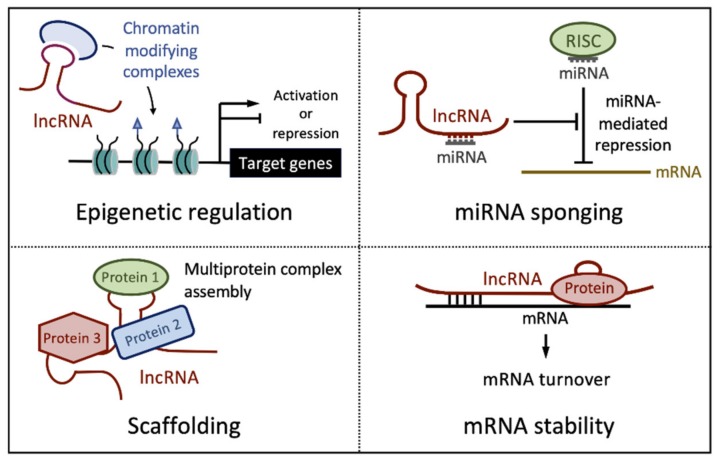
Major mechanisms of action of long noncoding RNAs (lncRNAs). See text for detail.

LncRNAs can interact with linear RNA or DNA sequences by base pairing. Moreover, secondary and tertiary lncRNA structures can also act as recognition surfaces for binding proteins with high affinity and specificity. Molecular interactions with RNA, DNA and proteins furnish almost endless possibilities for lncRNAs modes of action. These include epigenetic regulation of gene expression, forming scaffolds for macromolecular complex assembly, binding and inactivation of miRNAs (“sponging”), and regulating mRNA stability (Figure 1). 

The role of nuclear lncRNAs in epigenetic regulation has been extensively investigated, and there are numerous examples of lncRNAs affecting the epigenetic status of neighboring loci (in cis) or at distant loci (in trans). A classic example of a lncRNA acting in cis is the X-inactive specific transcript (XIST). During X-inactivation, XIST accumulates in cis where it tethers polycomb repressive complexes to silence genes on the X-chromosome, a phenomenon referred to as X-inactivation [19]. The HOX transcript antisense intergenic RNA (HOTAIR), transcribed from the HOXC locus, acts in trans by guiding chromatin repressive complexes to HOXD and other chromosomal loci [20]. Other lncRNAs such as the HOXA transcript at the distal tip (HOTTIP) and nettoie Salmonella pas Theiler’s (NeST) cause activation of target genes by recruiting WDR5, a component of the MLL/MLL1 histone H3 lysine 4 methyltransferase complex, which marks genes for transcriptional activation [21,22]. 

The ability of nuclear or cytoplasmic lncRNA to associate with proteins allows them to function as scaffolds for the assembly of individual proteins into functional complexes. The nuclear enriched abundant transcript 1 (NEAT1) lncRNA, for example, forms a complex with the HEXIM1 protein to assemble a complex that contains DNAPK, cGAS, TBK1 and IRF3, which is necessary to trigger innate immune signaling in response to cytoplasmic DNA sensing [23]. 

Cytoplasmic lncRNAs have also been reported to act as “miRNA sponges”. By base pairing with individual microRNAs they can restrain their abilities to bind to and inhibit their mRNA targets, thereby interfering with miRNA mediated repression [24]. 

Lastly, cytoplasmic lncRNAs can directly or indirectly bind mRNAs thereby modulating their stability and/or translation. The pro-differentiation terminal differentiation-induced lncRNA (TINCR), for example, binds and stabilizes mRNAs that are critical for critical for epithelial differentiation through the recruitment of the Staufen RNA binding protein [25]. 

Given the versatility of their biochemical modes of action, it comes as no surprise that cellular lncRNA expression is dysregulated in many cancers. However, there have been only very few studies that have carefully evaluated how specific, well established oncogenic drivers trigger dysregulation of lncRNA expression and how this may contribute to carcinogenesis. Given that HPV E6 and E7 are universal drivers of ~5% of human cancers, they are ideally suited to address this critical matter.

## 3. Deregulation of lncRNAs in Cervical Carcinomas

There have been numerous studies reporting increased (Table 1) or decreased (Table 2) expression of specific lncRNAs in HPV-associated premalignant lesions and cancers (see tables below for references). By proposing specific mechanisms of action and linking aberrant expression to specific oncogenic phenotypes, these studies suggest that dysregulated lncRNA expression may importantly contribute to HPV carcinogenesis by subverting cellular processes that have been referred to as “hallmarks of cancer” [7,26].

## 4. Deregulation of lncRNAs by HPV E6 and/or E7 Proteins

Several reviews have focused on the clinical implications of lncRNA expression changes in HPV-associated cancers [88,89,90], but dysregulation of cellular lncRNA expression in HPV-associated lesions and cancers does not infer that the observed changes represent a primary consequence of HPV infection and E6 and/or E7 expression. Some of the studies cited in the tables above implicated E6 and/or E7 as regulators of certain lncRNAs, including PVT1, MALAT1, SNHG12, lnc-CCDST, LINC01101 and LINC00277 [64,66,80,91,92] by depleting E6/E7 expression in cervical cancer lines. 

**Figure 2:**
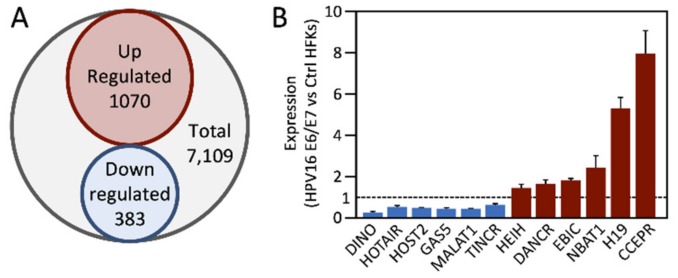
Differential expression of cellular lncRNAs in human papillomavirus (HPV)16 E6/E7 expressing human foreskin keratinocytes (HFKs). (**A**). Expression of annotated cellular lncRNA by RNA sequencing (RNAseq) analysis of HPV16 E6/E7 expressing and parental primary HFKs. (**B**). Quantitative reverse transcription PCR (qRT-PCR) analysis of select cellular lncRNAs in HPV16 E6/E7 expressing versus parental HFKs. See text for detail.

To determine how HPV16 E6/E7 expression deregulates lncRNA expression in normal human epithelial cells, we analyzed RNA sequencing (RNAseq) data of two independently derived populations of HPV16 E6/E7 expressing primary human foreskin keratinocytes (HFKs) and their donor and passage matched, control vector-transduced parental cells [93]. Of the 7109 annotated lncRNA species that were detectably expressed, the levels of 1453 was altered at least twofold. Of these, 1070 lncRNAs were expressed at higher levels whereas 383 were expressed at lower levels in HPV16 E6/E7 expressing HFKs than in parental HFKs (Figure 2A). 

From this list, we analyzed by quantitative reverse transcription PCR (qRT-PCR), expression of a small number of lncRNAs that were shown to be dysregulated in HPV-associated lesions and cancers (see Table 1; Table 2) or are well established modulators of cancer hallmarks targeted by HPV16 E6/E7. From this panel, the most significantly upregulated and downregulated lncRNAs are the cervical carcinoma expressed PCNA regulatory lncRNA (CCEPR) and the DNA damage-induced noncoding lncRNA (DINO), respectively. HOTAIR, human ovarian cancer-specific transcript 2 (HOST2), growth arrest-specific 5 (GAS5), metastasis associated lung adenocarcinoma transcript 1 (MALAT1) and tissue differentiation-inducing non-protein coding RNA (TINCR) were downregulated, whereas hepatocellular carcinoma up-regulated EZH2-associated lncRNA (HEIH), differentiation antagonizing non-protein coding RNA (DANCR), EZH2-binding lncRNA in cervical cancer (EBIC), neuroblastoma associated transcript 1 (NBAT1) and H19 were upregulated in HPV16 E6/E7 expressing HFKs (Figure 2B).

In the following some of the well-documented HPV16 E6/E7 regulated lncRNA species will be discussed in more detail. It will be important to determine whether similar results are also obtained with other high-risk HPV derived E6 and E7 proteins and in all the different cell types that high-risk HPVs are known to infect.

### 4.1. CCEPR (CCHE1)

Expression of the cervical carcinoma expressed PCNA regulatory (CCEPR) lncRNA (also referred to as cervical carcinoma high-expressed long non-coding RNA 1; CCHE1) is highly upregulated in cervical cancers and expression correlates with tumor size and poor prognosis of cervical cancer patients [32,94,95]. High level CCEPR expression has also been noted in other tumor types including osteosarcoma [96], uroepithelial bladder carcinoma [97], non-small cell lung carcinoma [98], hepatocellular carcinoma [99] and colorectal carcinoma [100]. Our own work revealed that CCEPR lncRNA is expressed at higher levels in HPV16 E6/E7 expressing HFKs than in parental HFKs [93]. Follow up studies revealed that CCEPR was upregulated in response to HPV16 E6 expression and this was independent of E6-mediatied TP53 degradation [33]. Consistent with previous findings [32], we found that CCEPR overexpression contributes to proliferation of cervical cancer cell lines. However, in contrast to this previous study our work provided no evidence for CCEPR increasing the levels of PCNA mRNA [33]. Moreover, we detected CCEPR was mostly in the nucleus [33]. Many nuclear lncRNAs contribute to gene expression via direct transcriptional and/or epigenetic regulation (Figure 1). Multiple alternative mechanisms of action including binding to ROCK1 or enhancing PAK2 expression through miR-922 sponging have been suggested for CCEPR from studies with other tumor types [101,102]. 

### 4.2. DINO

**Figure 3:**
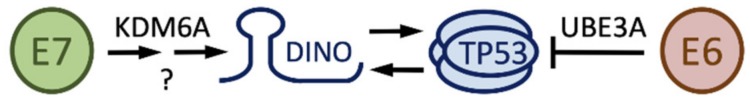
Regulation of damage induced noncoding (DINO) by HPV16 E6 and E7. See text for detail.

The TP53 responsive DNA damage induced noncoding (DINO) lncRNA (DINOL) binds and stabilizes TP53, thereby amplifying TP53-mediated signaling [103]. Our studies have shown that DINO was expressed at lower levels in HPV E6/E7 expressing HFKs than in control HFKs [93,104] (Figure 2B). Consistent with the ability of E7 to cause TP53 stabilization and E6 to target TP53 for degradation, we found that DINO levels were higher in E7 expressing HFKs but lower in E6 expressing HFKs than in parental cells [104]. We showed that E7 stabilizes TP53 through DINO and that E7 initially triggers DINO expression through a mechanism that involves epigenetic de-repression through the H3K27 demethylase KDM6A, which E7 is known to induce [105]. Once DINO expression is induced it activates TP53 which causes even higher DINO expression [104] (Figure 3). DINO depletion in E7 expressing cells renders cells less susceptible to cell death due to metabolic stress or treatment with DNA damage-inducing chemotherapy agents [104]. 

Given that cervical carcinoma cells retain wild-type TP53 expression [106], it is tempting to speculate that it may be possible to at least partially reconstitute the dormant TP53 tumor suppressor pathway in HPV-associated lesions and tumors by artificially modulating DINO levels and/or activity. Given that cancer cells cannot tolerate functional TP53, one might predict that such intervention may have valuable therapeutic benefits. 

### 4.3. HOTAIR

**Figure 4:**
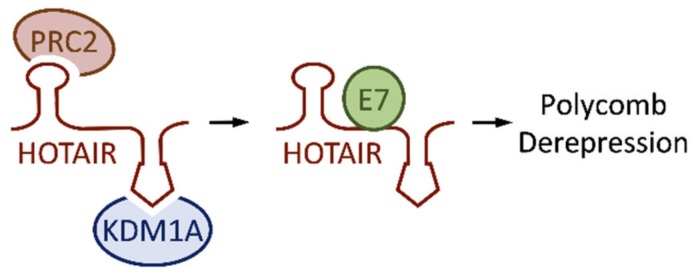
HPV16 E7 binding to HOX transcript antisense intergenic RNA (HOTAIR) may contribute to derepression of polycomb regulated genes by displacing HOTAIR bound KDM1A containing complexes and/or polycomb repressive complex 2 (PRC2); see text for detail.

HOTAIR is one of the best-studied lncRNAs in the context of human carcinogenesis. Expression is upregulated in many cancer types, suggesting that HOTAIR may be an oncogenic lncRNA (reviewed in [107]). HOTAIR was also reported to be highly expressed in cervical cancer tissues [44], but another study reported that HOTAIR levels were lower [79]. Our own analysis revealed lower HOTAIR levels in HPV16 E6/E7 expressing primary human keratinocytes as compared to parental cells [93] (Figure 2B). HOTAIR has been reported to recruit two distinct chromatin silencing complexes: polycomb repressive complex 2 (PRC2) at its 5’ end and histone lysine demethylase KDM1A (LSD1)-associated complexes at its 3’ end [108]. (Figure 4). HPV16 E7 was predicted to bind HOTAIR by in silico analysis, and this was validated by E7 immunoprecipitations followed by qRT-PCR analysis [79]. The authors speculated that E7 binding may impede the ability of HOTAIR to interact with PRC2 and/or KDM1A complexes (Figure 4). This may contribute to the ability of HPV16 E7 to cause de-repression of polycomb regulated genes [105,109] despite high level expression of the repressive H3K27 methyl transferase, EZH2 [110,111]. It will be interesting to determine whether there are additional biological consequences of the E7-HOTAIR interaction and if E7 can also form complexes with other lncRNAs.

### 4.4. EBIC (TMPOP2)

**Figure 5:**
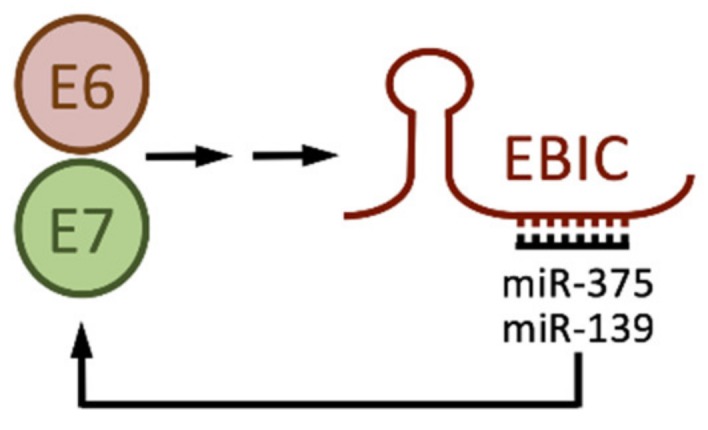
HPV E6 and E7 cause increased EZH2-Binding lncRNA in cervical cancer (EBIC) expression which, by sponging miR-375 and miR-139 causes increased E6 and E7 levels. See text for details and references.

The EZH2-Binding lncRNA in cervical cancer, EBIC, (also known as thymopoietin pseudogene 2, TMPOP2), may promote motility and invasion of cervical cancer cells by repressing CDH1 (E-Cadherin) expression though EZH2 [38]. Our own experiments revealed increased EBIC expression in HPV16 E6/E7 expressing HFKs as compared to control HFKs [93] (Figure 2B). Consistent with these results, a recent study suggested that EBIC expression was driven by E6/E7 expression, and largely due to E6 mediated TP53 degradation [39]. Interestingly, EBIC was reported to cause increased expression of HPV E6/E7 through a mechanism that involves sponging of miR-375 and miR-139, which have been previously reported to target HPV E6/E7 [112,113] (Figure 5). EBIC depletion in the HPV18 positive HeLa cervical carcinoma line inhibited proliferation by affecting the expression of cell cycle genes such as p21^CIP1^ (CDKN1A), cyclin E, and CDK2 [39]. 

### 4.5. H19

The onco-fetal lncRNA H19 is undetectable in most adult tissues but is re-expressed in a variety of tumors where it functions as an oncogenic driver [114,115]. H19 was reported to enhance cell proliferation and anchorage-independent growth of cervical cancer lines [42]. Our analysis showed that HPV16 E6/E7 expression in primary HFKs was sufficient to cause increased H19 expression [93] (Figure 2B) and a more recent study reported H19 expression is driven by HPV16 E6 [40]. The exact molecular mechanism by which HPV16 E6 drives H19 upregulation is unknown. 

The cancer cell-specific expression of H19 was harnessed to design a DNA therapy approach to selectively kill H19 expressing cancer cells by expressing diphtheria toxin A under the control of the H19 promoter. Such a plasmid, referred to as BC-819, is currently in phase II clinical trials for non-muscle invasive bladder cancer [116], has been tested in early phase trials for other tumor types [117,118] and has shown efficacy in preclinical animal models of a variety of other human tumors [119,120]. Moreover, transfections of HPV16 (CaSki) and HPV18 (HeLa, SW756) positive cervical cancer lines with an H19 promoter driven diphtheria toxin A expression vector inhibited their proliferation [121], suggesting that BC-819 or a similar reagent may also show therapeutic efficacy in HPV16 and HPV18 positive lesions and cancers.

This approach highlights how properties of lncRNAs can be exploited to develop therapeutics without directly targeting a lncRNA or without a detailed understanding of the upstream regulators. 

### 4.6. FAM83H antisense RNA 1 (FAM83H-AS1)

The FAM83H antisense RNA 1 (FAM83H-AS1) lncRNA is upregulated in many types of cancer, including cervical cancers, and a high level of FAM83H-AS1 expression correlates with poor survival [40]. FAM83H-AS1 expression is driven by E6 and is independent of TP53 degradation and may involve p300. FAM83H-AS1 is nuclear and depletion in cervical cancer cell lines inhibits proliferation and migration and causes apoptosis [40].

### 4.7. DANCR and TINCR

**Figure 6:**
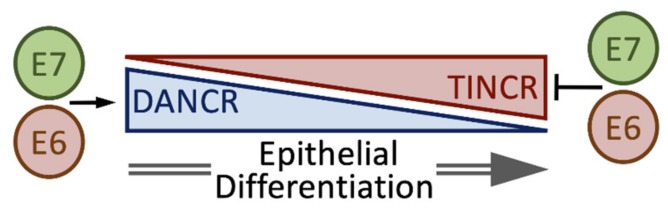
Regulation of keratinocyte differentiation by differentiation antagonizing non-protein coding RNA (DANCR) and tissue differentiation-inducing non-protein coding RNA (TINCR). See text for details.

LncRNAs are key regulators of epidermal differentiation. Both a pro-differentiation lncRNA, (TINCR) [25], and an anti-differentiation lncRNA, (DANCR) [122], have been described (Figure 6). 

Our studies revealed that TINCR levels were reduced in HPV16 E6/E7 HFKs, while DANCR levels were increased [93] (Figure 2B). HPVs are well known to alter epithelial cell differentiation and one mechanism, inhibition of the non-receptor protein tyrosine phosphatase, PTPN14, by HPV E7 proteins through UBR4 mediated degradation has recently been elucidated [123]. Nonetheless, it is conceivable that E6 and/or E7 may regulate epithelial differentiation at least in part by modulating TINCR and/or DANCR expression. DANCR levels were shown to be elevated in cervical tumors [36,37] and TINCR levels were lower [84]. Depletion of DANCR in cervical cancer cell lines blunted proliferation, migration, and invasion [37]. Whether this effect is related to DANCR’s ability to inhibit differentiation remains to be determined.

### 4.8. Colorectal Neoplasia Differentially Expressed (CRNDE) lncRNA

The colorectal neoplasia differentially expressed (CRNDE) lncRNA is overexpressed in cervical cancer tissues [34,35] and correlates with tumor size and poor clinical outcome. [35]. Our RNAseq results suggest that CRNDE overexpression is driven by HPV16 E6/E7 expression [93]. CRNDE overexpression in cervical cancer lines caused increased proliferation and tumorigenicity in xenograft assays [35]. Conversely, CRNDE depletion in cervical cancer lines inhibited migration and invasion and reduced tumorigenicity in xenograft assays. A range of CRNDE downstream targets and mechanisms of action have been proposed. One study proposed that CRNDE drives cervical cancer growth by inhibiting expression of the TP53 regulated apoptosis modulator, PUMA [35], whereas another study reported that CRNDE overexpression resulted in increased cyclin B1 expression through miR-183 sponging [34]. These two mechanisms are not mutually exclusive and suggest that CRNDE may be an important modulator of the HPV16 E6/E7 oncogenic drivers.

### 4.9. Maternally Expressed Gene 3 (MEG3)

The maternally expressed gene 3 (MEG3) lncRNA is expressed at lower levels in cervical cancer tissues than in normal cervical epithelium. Low MEG3 expression was correlated with tumor size, the presence of lymph node metastases and HPV expression [82]. Our RNAseq data suggest that decreased MEG3 expression is a direct consequence of HPV E6/E7 expression [93]. Ectopic MEG3 expression in cervical cancer lines inhibited proliferation, increased apoptosis and reduced tumorigenicity in xenograft models [81]. Consistent with the ability of MEG3 to activate TP53 [124], these effects were at least in part mediated by TP53 activation through miR-21-5p [82]. A later study by the same group suggested a different or additional mechanism and provided evidence that MEG3 may affect tumorigenicity by binding and targeting phospho-STAT3 for proteasomal degradation.

## 5. Concluding Remarks

**Figure 7:**
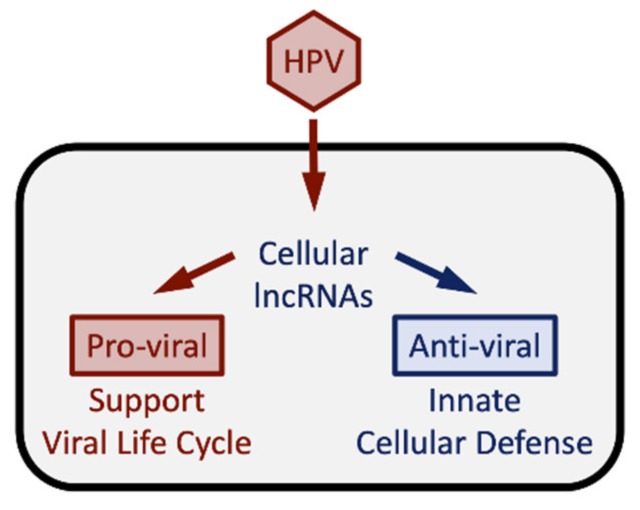
Alterations in lncRNA expression induced by HPV infection reflect the full spectrum of the molecular arms race of the virus and the host cells and may be different for specific HPV types. See text for details.

Noncoding genes have long been belittled as “junk” DNA and their expression has been considered “transcriptional noise”. However, some of these noncoding RNAs, particularly miRNAs, circular RNAs (circRNAs) and lncRNAs, are now recognized as regulators of a variety of cellular processes. Given that viruses need to reprogram their host cell to establish and maintain persistent infections and to support the synthesis of viral progeny, it is thus not surprising that the cellular transcriptome of non-coding genes is altered in virally infected cells. Indeed, several reports have indicated that viral infections cause substantial alterations of the expression profile of host cellular lncRNAs [125,126,127]. The host cellular lncRNA transcriptome changes in response to a viral infection reflect the “molecular arms race” between the intruding virus and the host’s defense responses [6,128]. They include “pro-viral” lncRNAs that the virus engages to invade and reprogram the host cell in order to support the viral life cycle, and “anti-viral” lncRNAs that the cell triggers as part of the arsenal of innate and adaptive responses against the unfriendly takeover attempt by the virus (Figure 7). 

Like other viruses, HPVs dramatically remodel the host cellular lncRNA transcriptome [125,126,127]. While it is technically straightforward to catalogue these changes, it remains challenging to mechanistically comprehend their biological consequences. Upstream regulators of lncRNAs can be determined by the same experimental approaches that have been developed for protein coding mRNAs. Similarly, depletion or overexpression experiments followed by determining transcriptional or biological readouts can offer vital clues regarding the molecular pathways that specific lncRNAs are involved in, provided that such experiments are performed in biologically relevant cell types. Further, experiments where TP53 and/or RB1 are depleted may help to distinguish between changes in cellular lncRNA expression that are caused by TP53 and/or RB1 loss and those that reflect other mechanisms of the E6 and/or E7 proteins. Moreover, it will be interesting to determine changes in cellular lncRNA expression in response to expression of the full HPV genomes, since these changes may be different than upon E6/E7 expression as is observed in HPV-associated tumors. Since there is often limited sequence homology in lncRNAs between different species, it is difficult to use animal models to study the relevance of these lncRNA changes. Hence, relevant cell-based models and organoid models are best suited to address these issues. Lastly, given the versatility of lncRNAs to function as molecular matchmakers for nucleic acids as well as proteins in the nucleus and/or the cytoplasm (Figure 1) it remains challenging to accurately and conclusively determine their molecular mechanisms of action. 

Viral proteins may also alter the biological activities of lncRNAs by direct or indirect binding, thereby changing their protein and/or nucleic acid interactomes. As described in Section 4.3., HPV16 E7 has been reported to interact with HOTAIR, thereby potentially impeding its ability to repress polycomb regulated genes [79]. Given that HPV16 E7 and E7 have been reported to interact with a large number of protein targets [9,10], it is conceivable that they may also have multiple lncRNA interaction targets.

Studies with oncogenic viruses have been crucial for the discovery of oncogenes and tumor suppressors and helped establish foundational concepts regarding their modes of action. Mechanistic evaluation of lncRNAs as oncogenic drivers in the context of viral carcinogenesis promises to provide similarly important insights. 

Some lncRNAs may serve as biomarkers for cancer detection, metastasis and survival of patients [129], and the prostate cancer specific lncRNA, prostate cancer antigen 3 (PCA3) is an FDA approved lncRNA biomarker for prostate cancer detection [130]. The EGFR-AS1 lncRNA is a predictor of the therapeutic response to erlotinib, an anticancer drug that targets epidermal growth factor receptor (EGFR), in that erlotinib-resistant lung cancer cell lines expressed very low levels of EGFR-AS1 [131]. Erlotinib combined with cisplatin and radiotherapy showed promising results in phase 2 clinical trials for cervical cancers [132]. EGFR-AS1 levels were lower in HPV16 E6/E7 expressing cells than in control cells [93]. Hence, EGFR-AS1 expression may serve to identify cervical cancer patients likely to benefit from erlotinib therapy. 

Oncogenic or tumor suppressive lncRNA are excellent candidates for direct therapeutic targeting and lncRNA-based therapeutics can be developed through a variety of approaches. Oncogenic and pathogenic lncRNAs can be depleted by RNAi or RNAse H-mediated decay by antisense oligonucleotides (ASOs). On the other hand, tumor suppressive lncRNAs can be introduced in tumors by gene therapy methods or nanoparticle delivery. Furthermore, similar to miRNAs, lncRNA activities can be modulated by nucleic acid-based mimics and inhibitors. Once considered undruggable, RNAs, including lncRNAs are now considered druggable with small molecules [133,134,135].

The concept that lncRNAs can be used to specifically kill virally infected cells has been demonstrated by the selective killing of HIV-1 infected macrophages upon depletion of a lncRNA upregulated by HIV-1 infection, SAF (FAS-AS1) [136]. Therefore, studies aimed at identifying vulnerabilities of HPV-infected cells to inhibiting specific lncRNAs are clearly warranted and may provide lncRNA targets to be exploited for developing HPV-specific therapeutic approaches.

Lastly, the regulatory elements of lncRNAs that are highly expressed in cancers may be harnessed for therapeutic approaches as in the case of BC-819, the vector driving expression of the diphtheria toxin A from the H19 promoter, which is in clinical trials and may be applicable to HPV16 positive tumors as well (see Section 4.5).

## Figures and Tables

**Table 1:** lncRNAs reported to be upregulated in various models of cervical lesions and cancers.

lncRNA	Oncogenic Phenotype	Proposed Mechanism	References
ANRIL	Proliferation, migration, invasion	PI3K/AKT; Cyclin D1, CDK4, CDK6, N-cadherin, Vimentin expression	[27,28]
ARAP1-AS1	Proliferation, invasion	MYC translation by PSF/PTB	[29]
BLACAT1	Proliferation, migration, invasion	WNT signaling/β-catenin	[30]
CCAT2	Proliferation, apoptosis	None reported	[31]
CCEPR (CCHE1)	Proliferation	PCNA mRNA stabilization	[32]
	Proliferation	independent of PCNA mRNA	[33]
CRNDE	Proliferation, migration, invasion	miR-183 sponging/cyclin B1	[34]
	Proliferation	PUMA expression	[35]
DANCR	Proliferation, migration, invasion	miR-665 sponging/TGFβ-R1-ERK-SMAD	[36]
	Proliferation, migration, invasion, epithelial to mesenchymal transition (EMT)	miR-335-5p sponging/ROCK1	[37]
EBIC (TMPOP2)	Motility, invasion	E-cadherin silencing by EZH2	[38]
	Proliferation	miR-375, miR-139 sponging HPV E6/E7 expression	[39]
FAM83H-AS1	Proliferation, migration and apoptosis	G1/S-phase transition	[40]
GATA6-AS	Migration, invasion	MTK-1	[41]
H19	Proliferation, anchorage independent growth	None reported	[42]
HOTAIR	Apoptosis, invasion, migration	NOTCH signaling	[43]
	Apoptosis, proliferation, invasion	miR-23b sponging/MAPK1 axis	[44]
	Autophagy, EMT	WNT signaling	[45]
	Proliferation	miR-143-3p sponging/BCL2	[46]
HOXD-AS1	Proliferation	Ras/ERK	[47]
Linc00483	Proliferation, apoptosis, invasion, migration	miR-508-3p sponging/RGS17	[48]
LINP1	DNA damage repair (Non-homologous end joining)	KU80, DNA-PKcs binding	[49]
Lnc-IL7R	Apoptosis	BCL2/caspase 3	[50]
LUCAT1	Proliferation, migration, invasion	miR-181a sponging	[51]
MALAT1	Cell invasion and metastasis	inhibition of EMT genes	[52]
	Proliferation, migration, invasion	miR-625-5p/AKT2	[53]
	Proliferation	Mir-625-5p/NF-kB signaling	[54]
	Cisplatin resistance	PI3K/AKT	[55]
MIR205HG	Proliferation, apoptosis, migration	SRSF1/KRT17 axis	[56]
NEAT1	Proliferation, invasion	PI3K/AKT	[57,58]
	Colony formation, migration, invasion	miR-133a sponging/SOX4	[59]
NORAD	Proliferation, invasion	miR-590-3p sponging/SIP1	[60]
PANDAR	Proliferation	None reported	[61]
PVT1	Proliferation, invasion	Inhibiting TGFβ; miR-140-5p sponging/SMAD3	[62,63]
	EMT, chemoresistance	miR-195 epigenetic silencing	[64]
SNHG8	Proliferation, apoptosis	RECK silencing by EZH2	[65]
SNHG12	Proliferation, apoptosis	ERK/Slug	[66]
SNHG16	Proliferation, invasion	PARP9 expression by SPI1 binding	[67]
TUG1	Proliferation, apoptosis, invasion, tumor growth	miR-138-5p sponging/SIRT1	[68]
	Proliferation, apoptosis, EMT	BCL-2, caspase 3; fibronectin, vimentin, and cytokeratin	[69]
TP73-AS1	Proliferation, migration	miR-329-3p sponging/SMAD2	[70]
	Proliferation, migration, invasion	miR-607 sponging/CCND2	[71]
UCA1	Radioresistance	HK2/glycolytic pathway	[72]
XIST	Proliferation	miR-140-5p sponging/ORC1	[73]
	Proliferation, invasion, apoptosis, EMT	miR-200a sponging/FUS	[74]
ZEB-AS1	Proliferation, migration, invasion, EMT	ZEB1 expression	[75]

**Table 2:** lncRNAs reported to be downregulated in various models of cervical lesions and cancers.

lncRNA	Oncogenic Phenotype	Proposed Mechanism	Reference
GAS5	Proliferation, invasion, migration	E-cadherin, Vimentin	[76]
	Proliferation, migration, invasion, colony formation	miR-21 expression/STAT3	[77]
	Radiosensitivity	miR-106b sponging/IER3	[78]
HOTAIR	Decreased polycomb repression	Binding to HPV E7	[79]
Lnc-CCDST	Migration, invasion, angiogenesis	DHX9, MDM2 scaffolding	[80]
MEG3	Proliferation, apoptosis	Binding, degradation of P-STAT3	[81]
	Proliferation, colony formation, apoptosis	miR-21-5p expression/TP53	[82]
STXBP5-AS1	Viability, invasion	miR-96-5p expression/PTEN	[83]
TINCR	Differentiation, colony formation, migration	S100A8 and other ZNF750 targets	[84]
WT1-AS	Proliferation	TP53	[85]
	Proliferation, invasion, migration	miR-203a-5p binding/FOXN2	[86]
XLOC_010588	Proliferation	MYC mRNA binding/degradation	[87]
